# Politicization and Polarization in COVID-19 News Coverage

**DOI:** 10.1177/1075547020950735

**Published:** 2020-10

**Authors:** P. Sol Hart, Sedona Chinn, Stuart Soroka

**Affiliations:** 1 University of Michigan, Ann Arbor, MI, USA; 2 University of Wisconsin-Madison, Madison, WI, USA

**Keywords:** COVID-19, politicization, polarization, media coverage, risk communication

## Abstract

This study examines the level of politicization and polarization in COVID-19 news in U.S. newspapers and televised network news from March to May 2020. Using multiple computer-assisted content analytic approaches, we find that newspaper coverage is highly politicized, network news coverage somewhat less so, and both newspaper and network news coverage are highly polarized. We find that politicians appear in newspaper coverage more frequently than scientists, whereas politicians and scientists are more equally featured in network news. We suggest that the high degree of politicization and polarization in initial COVID-19 coverage may have contributed to polarization in U.S. COVID-19 attitudes.

In late 2019, a novel coronavirus, COVID-19, began to spread throughout the world. COVID-19 was declared a public health emergency of international concern by the World Health Organization on January 30 and a pandemic on March 11, 2020 (World Health Organization, 2020). The infection rate and death toll have been substantial; by the end of May 2020, at least 6 million people had been infected and at least 369,000 had died globally (Beaumont et al., 2020). Many of the infections have been concentrated in the United States, with at least 1.8 million individuals infected and 100,000 individuals killed by COVID-19 in the United States by the end of May (Centers for Disease Control and Prevention, 2020; Johns Hopkins University of Medicine, 2020).

While COVID-19 poses a significant risk, political responses and public perceptions in the United States have been divided across political ideological lines (Milligan, 2020; Roberts, 2020). This raises questions about the role that both politicians and the media have played in amplifying politicization and polarization of COVID-19, as this kind of news coverage can influence public attitudes in ways that exacerbate partisan divides (Bolsen et al., 2014; Brulle et al., 2012; Druckman et al., 2013). Examining the first months of COVID-19 news coverage may therefore help us to better understand what informed the public’s initial perceptions of COVID-19. Though research to date has not examined politicization and polarization in COVID-19 news coverage, recent research by Chinn et al. (2020) investigating politicization (the degree that politicians are mentioned in conjunction with the issue) and polarization (how discussion varies based on the presence of actors from different political parties) in climate change news coverage offers a useful methodological approach for analyzing these features in news content. We draw on this approach in the present study, which uses both dictionary and unsupervised machine learning methods to investigate the degree to which newspaper and network news coverage of COVID-19 was polarized and politicized during the first 3 months of heightened news coverage (March, April, and May 2020).

## Background

The global increase in COVID-19 infections through early 2020 led the United States to declare COVID-19 a national emergency on March 13 (The White House, 2020), and the majority of states had issued stay-at-home orders by the end of March 2020 (KFF, 2020).

While there has been broad public agreement for some preventative measures, such as restricting international travel to the United States, closing K-12 schools, and canceling major sports and entertainment events (Van Green & Tyson, 2020), Americans have been divided in their perceptions of the government response, confidence in scientists, and support for protective actions. For example, 83% of Republicans rate President Trump’s response to COVID-19 as good or excellent, whereas only 18% of Democrats do so (Van Green & Tyson, 2020). In addition, the public is polarized on perceptions of scientists and actions to respond to the pandemic. While in 2019 Democrats had greater confidence than Republicans that both medical scientists and scientists in general would act in the best interests of the public, this difference dramatically widened in April 2020, especially with respect to medical scientists, as Democratic confidence increased while Republican confidence remained flat (Funk et al., 2020). With regard to protective actions, a minority of Republicans, compared to a majority of Democrats, felt that social distancing was helping a lot to slow the spread of coronavirus, that there was insufficient testing for coronavirus, and that more people needed to follow social distancing guidelines (Funk et al., 2020).

These partisan differences in public opinion correlate with observed behavioral differences. Analyses using GPS data from smartphones found that areas with more Republicans exhibited less social distancing than those with more Democrats (Allcott et al., 2020). Several other studies corroborate that Democrats are more likely to comply with social distancing guidelines (Goldstein & Wiedemann, 2020; Kushner Gadarian et al., 2020; Painter & Qiu, 2020), while Republicans and individuals with greater faith in President Trump are less likely to do so (Graham et al., 2020).

Partisans’ perceptions of COVID-19 media coverage are starkly polarized as well. Partisans are dramatically divided on whether news media coverage of COVID-19 is accurate (Dem: 66%, Rep: 31% agree), working for the benefit of the public (Dem: 66%, Rep: 28% agree), helping the country (Dem: 63%, Rep: 27% agree), and getting people the information they need (Dem: 73%, Rep: 44% agree; Gottfried et al., 2020). Recent polling revealed that partisans are more polarized on whether journalists will act in the best interests of the public (Rep: 23%, Dem: 70% agree) than any other group (e.g., university professors or business leaders; Gottfried et al., 2020).

Differences in partisans’ perceptions of COVID-19 may be due, in part, to differences in partisan elites’ messaging on the issue. President Trump (Franck, 2020) and leading conservative political commentators (Peters & Grynbaum, 2020; Rupar, 2020) frequently referred to COVID-19 as a “hoax” and have been dismissive of the risks the virus posed. Right-wing media outlets were more likely to spread misinformation about COVID-19 in the beginning of the outbreak, and more frequent viewers of conservative media outlets were more likely to be misinformed about COVID-19 (Motta et al., 2020). Given these trends in partisan messaging, a critical question is the degree to which mainstream news outlets amplified the voices of political actors in COVID-19 coverage, as well as the extent to which language in news coverage highlighted partisan differences when discussing Republican and Democratic actors.

The current study speaks to these issues by investigating the degree to which mainstream newspaper and network TV news coverage on COVID-19 was politicized and polarized between March and May 2020. Politicization in news coverage of an issue refers to the prominence of political actors in coverage (Bolsen et al., 2014; Chinn et al., 2020). An issue may become politicized for a number of reasons, and politicization is not inherently negative. For example, coverage of politicians coming together to address a social risk is likely to be highly politicized. But biases in newsroom norms and the desire to draw audience attention to a story can also lead to greater politicization of content. Journalists often use personalized stories focusing on arguments between competing actors to highlight conflict and dramatize issues (Bennett et al., 2007; Boykoff & Boykoff, 2007; Feldman et al., 2015; Hart & Feldman, 2014). Personalized, dramatic coverage often features leading politicians who serve as representatives of competing political camps (Bennett et al., 2007; Wilkins & Patterson, 1987). This kind of politicized coverage can influence public views, such that individuals may rely on political leaders more than on scientists when forming impressions of an issue (Bolsen et al., 2014; Slothuus & de Vreese, 2010). For science and risk issues, such as COVID-19, it is therefore beneficial to examine the degree to which both politicians and scientists are featured in news in order to determine how much emphasis is placed on scientific and political perspectives (Chinn et al., 2020).

A high degree of politicization may be more troubling when coverage is also highly polarized, that is, highly differentiated along partisan lines. For a novel issue, such as a new pandemic, the news media is typically the primary way the public learns about the issue (Kasperson et al., 1988). When such coverage is both highly politicized and polarized, motivated reasoning (Taber et al., 2009) and a predisposition of the public to rely on political over scientific views (Bolsen et al., 2014; Slothuus & de Vreese, 2010) mean that news coverage can amplify partisan differences in risk perceptions and responses to an issue. That is, when media coverage is polarized, members of the public are likely to form opinions in line with political elites they trust and reject information not aligned with this view, even if the information comes from experts (Druckman et al., 2013). Thus, the degree to which media coverage of pandemics, like COVID-19, is both politicized *and* polarized is a critical research question. While a number of studies have looked at various factors of how news media cover pandemics (Dudo et al., 2007; Klemm et al., 2016; Lee & Basnyat, 2013; Vasterman & Ruigrok, 2013), they have not looked at politicization and polarization in such coverage. Thus, our investigation of politicization and polarization makes a novel contribution to the study of media coverage of pandemics and is important for understanding a likely factor contributing to the rapid polarization around COVID-19 in the United States.

In sum, media coverage plays a critical role in shaping public opinion around emerging science and risk issues, and the degree of politicization and polarization of such news coverage may be important and influential factors. The nature of politicization and polarization has been examined in other science issues, such as global climate change (Boykoff & Boykoff, 2007; Chinn et al., 2020; Chinn & Pasek, 2020; Feldman et al., 2015; Fisher et al., 2013; Guber, 2013; Hart & Feldman, 2014), agricultural biotechnology (Maeseele, 2011), health issues such as HPV vaccines and mammography screening (Fowler & Gollust, 2015), GMOs (Mintz, 2017), and additional controversial science issues (Drummond & Fischhoff, 2017). However, there is not yet work on the prevalence these features in COVID-19 news coverage. In the present study, we examine the degree to which newspaper and network news coverage of COVID-19 between March and May 2020 was politicized and polarized, as well as the frequency with which political actors were represented in news stories compared to scientists.

## Method and Results

### Data

Our data set includes all morning and evening news broadcasts, as well as newsmagazine-format programs, from ABC, CBS, and NBC, and all front-section stories from six regional and national newspapers: *USA Today, The Washington Post, The Philadelphia Inquirer, The New York Times, The Los Angeles Times, The Minneapolis Star-Tribune*, and *The Atlanta Journal-Constitution* from January through May 2020. These data were collected from Lexis-Nexis. This initial database includes 36,620 stories.

From this data set, we identified articles and broadcasts about COVID-19. We did so by identifying articles and broadcasts that mentioned “covid,” “coronavirus,” or “corona.” In order to limit our analyses to stories that had substantive coverage of COVID-19, we restricted our analysis to stories in which a COVID-19 keyword was mentioned at least three times. Before early March, 10% or fewer of daily stories focus on COVID-19; the number of stories from this early period is likely too few to produce meaningful results with the content-analytic methods used in this study. By mid-March, however, over 40% of coverage was focused on COVID-19. We accordingly focus on coverage from March 1 until May 26, the day after George Floyd was killed (and consequently the point at which news content shifts to other pressing issues). This database includes 22,111 stories, which included 13,820 stories that mention COVID-19 at least once and 6,985 stories that mention COVID-19 at least three times. As mentioned above, we focused on stories with substantive coverage, for the analyses presented here we drew on only the 6,985 stories in the database that mention COVID-19 at least three times.

The selection of articles and all subsequent analyses were conducted using the quanteda package in R (Benoit et al., 2018). As different methods were used for the politicization and polarization analyses, for clarity we first discuss the methods and results for politicization and then present the methods and results for polarization.

### Politicization

Our analysis of politicization of COVID-19 follows the approach adopted by Chinn et al. (2020), in which politicization is measured by the frequency that news articles mention political actors. Chinn et al. utilized a dictionary-based approach to examine a corpus of 30 years of data; the large time frame made it necessary to use general dictionaries to capture mentions of political actors, with words like “Republican,” “conservative,” “Democrat,” and “progressive,” as named political actors change over time. In the limited time scale of the present analysis (March-May 2020), there is likely to be more consistency in named political actors. This affords the opportunity to build from the general dictionaries used by Chinn et al. (2020) to add targeted dictionaries to capture mentions of prominent politicians such as Trump, McConnell, and Biden. Table 1 shows Democrat and Republican dictionaries separately; note that they are combined for this analysis.^1^

**Table 1.** Dictionaries.

Dictionary	Words
Covid-19	“corona,””coronavirus,””covid”
Scientist	[“scientist,^*^” “research,^*^” “professor^*^”], “health official,^*^” “doctor,^*^” “dr, “ “health commission,” “expert,^*^” “health leader,^*^” “health service,^*^” “health authorit,^*^” “world health organization,” “centers for disease control and prevention,” “cdc,” “national institutes of health,” “health and human services,” “mayo clinic,” “johns hopkins,” “fauci,” “birx,” “tedros”
Republican	[“republican,^*^” “gop,” “conservative^*^”], “trump,” “pence,” “mcconnell,” “white house,” “administration”
Democrat	[“democrat,^*^” “liberal,^*^” “progressives”], “pelosi,” “schumer,” “biden,” “obama,” “newsom,” “whitmer,” “cuomo,” “biden,” “sanders”

*Note.* Words in brackets make up the general dictionary developed by Chinn et al. (2020). The targeted dictionary includes these words and adds the additional words listed by category.

Using the raw count of politicians allows us to efficiently measure politicization in large data sets, but it is important to note that not all aspects of politicization are captured. For example, some actors, including political activists, are not captured by the dictionary. In addition, journalistic choices that may have political implications, such as how an issue is framed, are not captured. Finally, while we have included the names of prominent politicians in the analysis, the analysis will also fail to capture mentions of less prominent political actors.

In addition to our measure of politicization, which captures the presence or absence of political actors, we examine the frequency with which scientists are mentioned in COVID-19 news stories. While a story may be politicized regardless of the presence or absence of scientists, the comparison of how often partisan actors and scientists are mentioned provides an indication of the degree to which news articles are focusing on scientific, as compared to political, aspects of an issue. As with the measure of politicization, we built from the general scientist dictionary developed by Chinn et al. (2020) by adding keywords relevant to COVID-19 (e.g., “health official”) as well as the names of prominent scientists (e.g., “Fauci” and “Birx,” see Table 1).

The total number of words identified in each article by the general and targeted politician dictionaries are correlated at *r* = .70, while the scientist dictionaries are correlated at *r* = .47. For the sake of comparison, we ran politicization analyses using both the general dictionaries developed by Chinn et al. (2020) and the COVID-19 targeted dictionaries.

**Figure 1.**
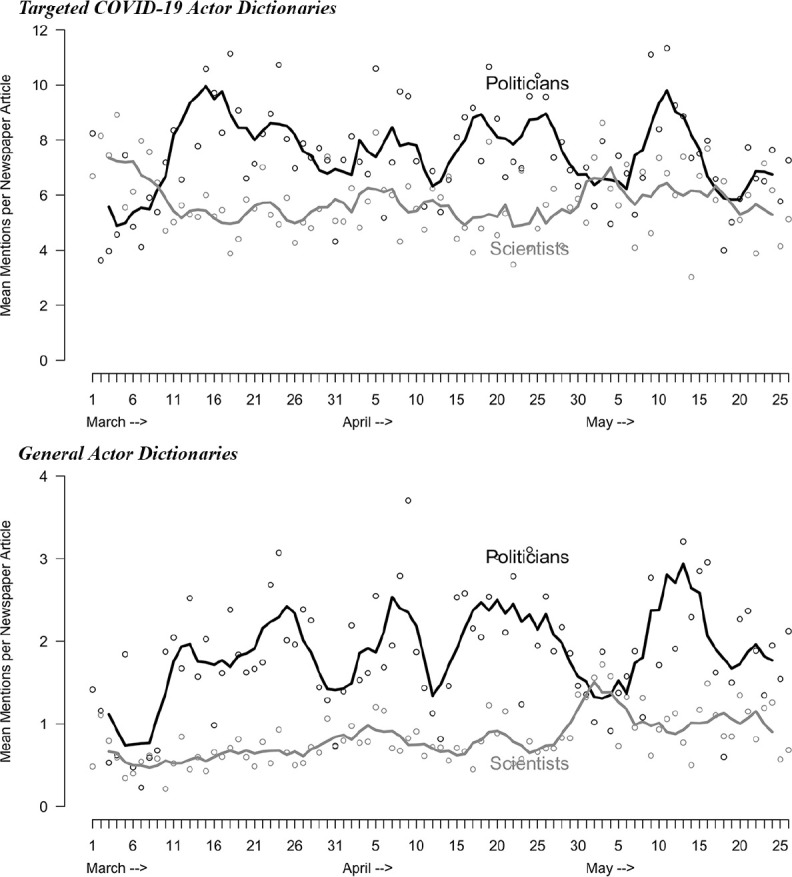
Politicization in newspaper coverage of COVID-19, March 1 to May 26, 2020. (A) Targeted COVID-19 actor dictionaries; (B) General actor dictionaries. *Note.* Lines represent centered 5-day moving averages. Dots represent actual data points for each day. The black lines and dots represent mentions of politicians and the grey lines and dots represent mentions of scientists.

Turning first to newspaper coverage, results using the targeted dictionaries are shown in the top panel of Figure 1; results using the general dictionaries are show in the bottom panel. Both suggest a similar pattern in newspaper coverage. Politicization increased substantially between March 6 and 13 and then remained elevated, albeit with some variation, through the end of May 2020. Both the general and targeted versions of the dictionaries also show that politicians received more mentions than scientists after mid-March.

The use of the general dictionary allows us to directly compare politicization in newspaper coverage of COVID-19 and politicization of newspaper coverage of global warming, analyzed by Chinn et al. (2020). It appears that COVID-19 newspaper articles, which have 1.59 mean mentions of politicians in newspaper articles over the time period examined here, are slightly more politicized than recent coverage of global warming, for which Chinn et al. found contained 1.33 mean mentions of political actors in newspaper articles in recent years. Note also that while global warming news coverage became gradually more politicized over many years (see Chinn et al., Figure 1), news coverage of COVID-19 saw a dramatic degree of politicization in newspaper coverage almost immediately.

**Figure 2.**
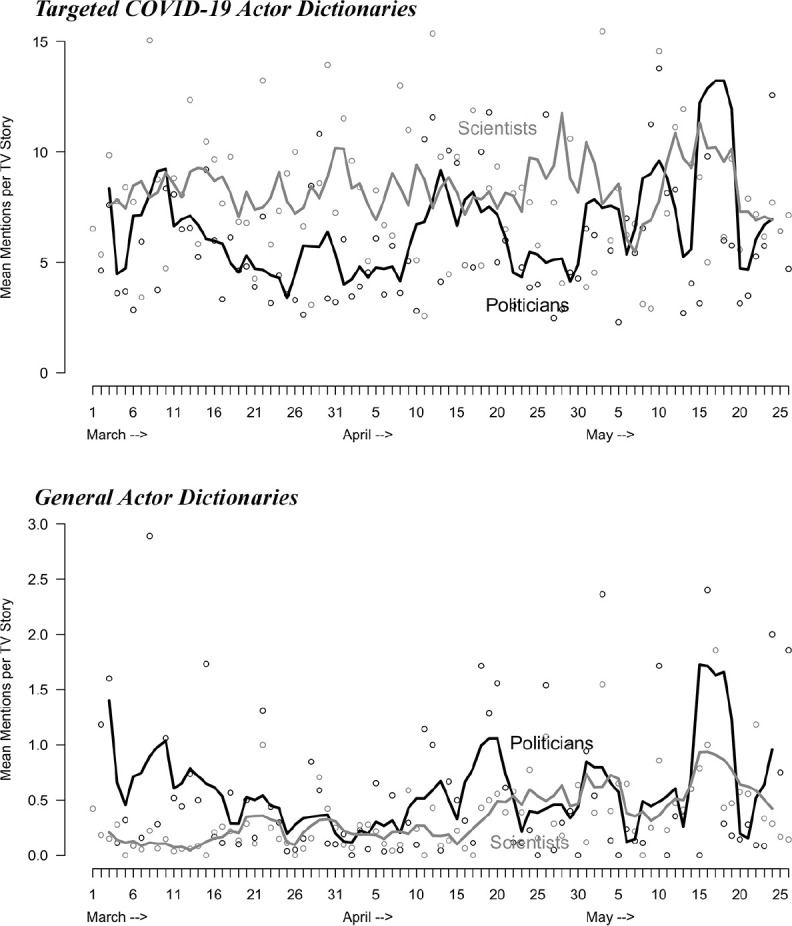
Politicization in network news coverage of COVID-19, March 1 to May 26, 2020. (A) Targeted COVID-19 actor dictionaries; (B) General actor dictionaries. *Note.* Lines represent centered 5-day moving averages. Dots represent actual data points for each day. The black lines and dots represent mentions of politicians and the grey lines and dots represent mentions of scientists.

The pattern of politicization is different when looking at network news coverage (see Figure 2). Neither the general nor the targeted dictionaries reveal a March increase in politicization. Rather, they show a somewhat consistent low level of politicization. When comparing the frequency of politician mentions to scientist mentions in network news coverage, the general dictionary reveals that politicians and scientists are mentioned at about the same rate, whereas the targeted dictionary finds more mentions of scientists than politicians in network news coverage.

### Polarization

We measure polarization using a similar approach as adopted by Chinn et al. (2020), relying on the unsupervised machine learning tool Wordfish (Slapin & Proksch, 2008). Wordfish estimates the likelihood of word mentions in each document relative to their frequency in other documents, and then assigns weights to words based on the degree to which those words distinguish documents across a single latent dimension, estimated through an iterative expectation-maximization algorithm. This approach has been used previously to examine differences between political actors in contexts such parliament speeches (Proksch & Slapin, 2010) and lobbying strategies used by interest groups (Klüver, 2011, 2012). The Wordfish procedure assigns each document a score on the latent dimension, and the degree to which those scores are correlated with partisanship is the measure of polarization that we focus on here. This measure quantitatively describes similarities or differences in the language surrounding Republican and Democrat mentions in COVID-19 articles.

We implemented Wordfish by extracting 200-word “windows” from COVID-19 articles that mention Republicans or Democrats (but not both).^2^ We then removed from those sentences any mention of the parties and first names so that these do not factor into our estimates of differences in language. These “cleaned” documents are the raw material examined with Wordfish. Once positions (scores on the latent dimension) were assigned by Wordfish to each document, we averaged the scores of all Republican documents and all Democrat documents, by month.^3^ The difference between the average position of Republican and Democrat COVID-19 texts serves as our measure of differences in language, with larger difference scores representing a greater degree of language polarization. To be clear: The dimension identified by Wordfish is an undefined latent dimension based on the language used in all documents, and the correlation between this dimension and partisanship indicates the degree to which the language surrounding Democratic mentions is different from the language surrounding Republican mentions.

**Figure 3.**
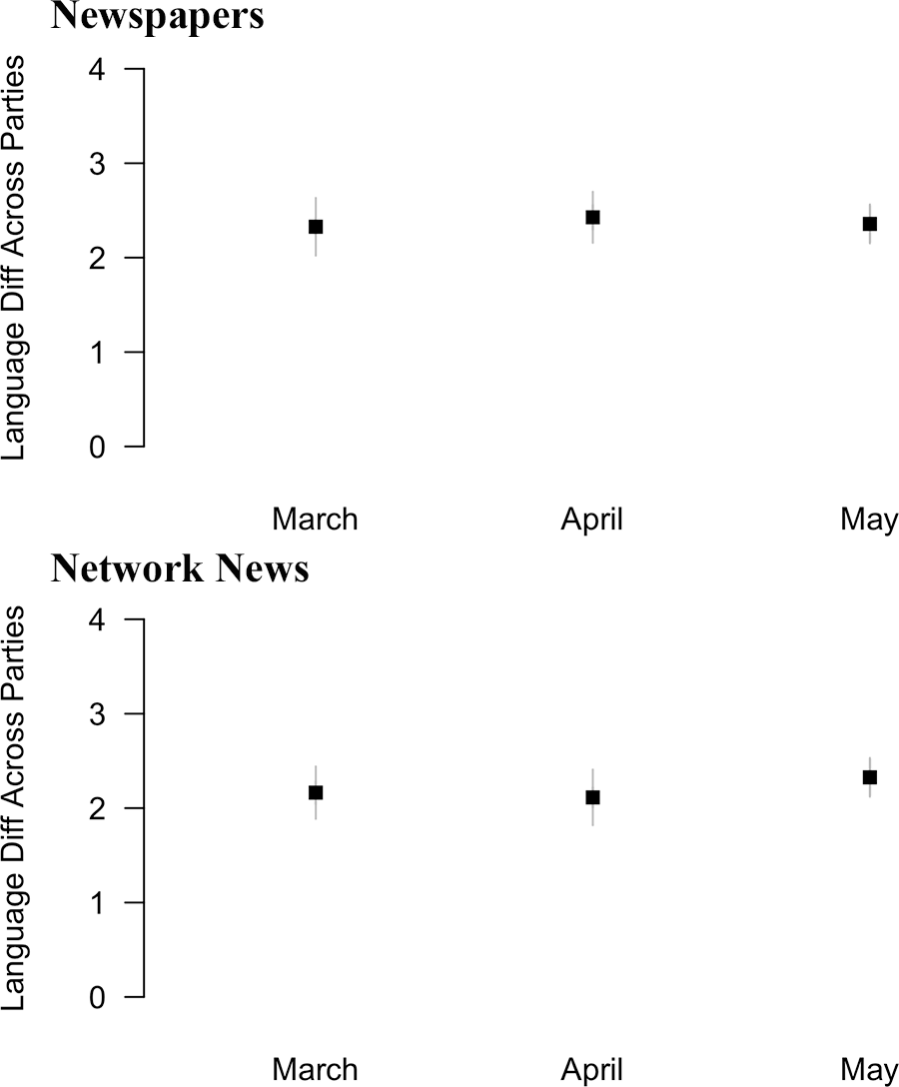
Polarization in COVID-19 coverage by month. (A) Newspapers. (B) Network news.

We separated newspaper and television documents for monthly analyses. Figure 3 shows the estimated difference in Wordfish-estimated scores between Democratic and Republican mentions in COVID-19 coverage for newspaper and television. Estimates are significantly different from zero in every case (standard errors are shown as grey bars in Figure 3), suggesting marked differences in the language used surrounding Democratic and Republican mentions in COVID-19 coverage between March and May 2020. There are not significant differences in polarization from one month to the next nor between newspapers and television.

**Figure 4.**
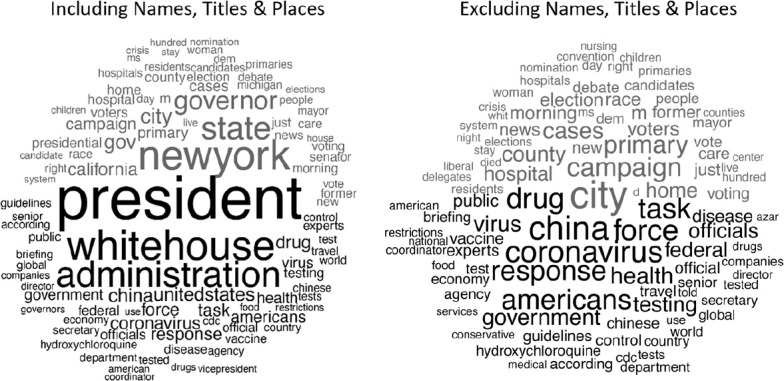
Words distinguishing Republicans and Democrats in COVID-19 coverage. (A) Including names, titles, and places; (B) Excluding names, titles, and places. *Note.* Black words in the bottom half of the comparison clouds are those most closely associated with Republicans and grey words in the top half are most closely associated with Democrats. The size of the words indicates both the frequency of use and distinctiveness of the word with the affiliated party.

Results in Figure 3 make clear that there are consistent differences in the language surrounding party mentions in news content. These differences appear to have been in evidence right from the start of the pandemic. It is therefore of substantive interest to identify the language that is driving the estimates in Figure 3. Figure 4 presents results from two “comparison clouds,” showing the top 100 words that most distinguish between Republican and Democratic texts (newspaper and television content is combined).^4^ Grey words distinguish Democratic documents; black words distinguish Republican documents. The comparison cloud in the left panel shows results using the data using for Figure 3. The cloud on the right replicates the analysis using data that additionally strips out last names, titles (e.g., president, governor) and places. Font size indicates both word frequency and the association of a word with the party—larger words are used more and are more distinctive to the party they are associated with.

The clouds make relatively clear that differences in language surrounding Republican and Democratic mentions are not clearly a function of markedly different discussions of policy and outcomes. Rather, the left cloud in Figure 4 highlights the degree to which polarization results in Figure 3 are driven by (1) national-level Republican actors versus (2) state- and local-level Democratic actors. That is, polarization in news stories about COVID-19 is most in evidence through the representation of dueling levels of government.

This is not to say that partisans did not have very different perspectives on COVID-19 concerns and policy, just that these are not the most prominent features of language differences between the two parties. Policy differences are slightly more evident in the right panel of Figure 4. These results suggest that Republican coverage is distinguished by language associated with federal responses to the novel coronavirus, alongside factors such as the need for a vaccine, China, and the (ultimately untrue) potential for hydroxychloroquine as a cure. Democratic coverage is distinguished by responses from Democratic governors, especially in New York and California; impacts on hospitals and residents; and consequences for the ongoing Democratic primaries.

Methodologically speaking, Figure 4 makes clear that the nature of our COVID-19 polarization measure is rather different than it was in the context of a long-standing climate change debate between politicians at a single level of government (Chinn et al. 2020). In the current instance, differences in journalistic language highlight the extent to which COVID-19 polarization has been driven by conflict between levels of government. This is polarization, to be sure, but it is perhaps relatively unique to this kind of highly federalized policy debate.

## Discussion

This study is the first to examine politicization and polarization in early news coverage of COVID-19. Overall, the analysis finds that newspaper coverage is highly politicized, network news coverage is somewhat less politicized, and both newspaper and network news coverage are highly polarized.

Looking first at newspaper coverage, the level of politicization in content increased very quickly around the time that a U.S. national health emergency was declared in March 2020, and remained elevated throughout the period of analysis. It is interesting to note that while Chinn et al. (2020) had found that mentions of scientists decreased while mentions of politicians increased in newspaper coverage of global warming, that pattern is not exhibited here. While political mentions quickly increase above those of scientist mentions, the frequency of scientist mentions remains fairly consistent throughout the period of analysis. Comparing the results found here to those found in Chinn et al. shows that levels of politicization and polarization in newspaper coverage of COVID-19 meet or exceed levels found in coverage of global warming, which is one of the most polarizing issues in the public eye (Leiserowitz et al., 2019).

The patterns in network news coverage of COVID-19 were somewhat different. Whereas polarization was high, similar to that found in newspaper coverage, politicization was lower. In addition, in contrast to newspaper coverage, using the general and targeted dictionaries yielded somewhat different results. The general dictionary revealed roughly equal levels of politician and scientist mentions, whereas the targeted dictionary found more scientist than politician mentions. It is not immediately clear why the pattern of coverage is different in newspapers and network news, or whether it will always be the case that the general and targeted dictionaries show similar patterns when analyzing newspaper coverage but different patterns when analyzing network news coverage; these are important questions for future research.

Taken together, the analyses suggests that (1) when looking at newspaper coverage, the general politicization dictionary serves as a reasonable proxy for a targeted analysis of an issue like COVID-19, although the dictionaries differ with respect to network news; (2) politicization is greater in newspaper than network news coverage; (3) the patterns of politicization are different in newspaper and network news coverage; (4) politicians have been mentioned more, relative to scientists, in newspapers than on network news; and (5) polarization is roughly even across news sources, meaning that while politicians are mentioned less in network news than newspaper coverage, mentions are still associated with highly polarized language.

The present study does not investigate what effects politicized and polarized media coverage has on public opinion. However, we know that politicized and polarized news coverage can influence public views and encourage individuals to follow political elites over experts (Bolsen et al., 2014; Brulle et al., 2012; Druckman et al., 2013). Signals of polarized views from opinion leaders, such as politicians, can cause individuals to fear social ostracization from their respective normatively influential groups if they express contrasting beliefs (Kahan, 2012). Thus, a high degree of politicization and polarization can create a polluted science communication environment (Kahan, 2012) that, combined with individual inclinations for motivated reasoning (Taber et al., 2009), amplifies value and belief differences on the issue. In line with these expectations, we saw that public opinion on COVID-19 was highly polarized (Gottfried et al., 2020; Van Green & Tyson, 2020) during the same time period that media coverage was highly politicized and polarized. Thus, it is likely that media coverage is contributing to the polarization of public attitudes, although experimental work examining how varying exposure to politicized and polarized COVID-19 news coverage influences public views is needed to confirm this.

A strength of the present study is the use of all COVID-19 news stories in network television news and a range of national and regional newspapers. This allows for a robust examination of politicization and polarization across the time period for analysis. Also, we were able to quantitatively compare politicization and polarization of COVID-19 coverage with previous analyses of global warming coverage (Chinn et al., 2020) to provide a referential context. It is also important to acknowledge several limitations in the present study. First, while our measure of politicization captures mentions of partisan actors, it is not able to speak to reasons why an issue may be politicized. That is, an issue could be politicized for a variety of reasons, including political scandals or bipartisan policy making, and the analytic approach adopted here is not able to distinguish between these. In addition, while we have created robust dictionaries that capture prominent political actors, any political actor not captured by our dictionary will not be included in the analysis, and therefore these results are likely to underestimate the degree of politicization in COVID-19 news coverage. Finally, while Wordfish offers a quantitative estimate of the magnitude of polarization, it does not provide information into the nature of such polarization. We therefore supplemented the Wordfish analysis with the comparison cloud in Figure 4 to better understand the nature of polarization in COVID-19 news coverage.

While media coverage of COVID-19 is dynamic and will likely shift in the months and years to come, the present study provides a robust examination of how initial coverage of COVID-19 was politicized and polarized. Comparisons between coverage of COVID-19 and coverage of climate change (Chinn et al., 2020) suggest that the first 3 months of substantive COVID-19 news coverage in the United States were at least as polarized and politicized as recent news coverage of global climate change, if not more so. While we do not offer guidance of how journalists ought to cover emerging science and risk issues, we raise the important note that this type of news coverage in the early months of COVID-19 is likely to amplify partisan differences in perceptions of the issue.

## Notes

1 Note that we do not capture titles of politicians (e.g., “Senator”) independent from mentions of political parties. Using both parties and titles risks the possibility that we double count some individuals. That said, this means that our measure of politicization will tend to slightly underestimate the total number of politician mentions.

2 We do so using the quanteda package in R. We exclude “windows” that include mentions of both parties because we cannot easily attribute those texts to one or the other party.

3 In March, there are 153 documents for newspapers and 65 for television; in April, 167 and 52; in May, 101 and 37.

4 “Distingushing” words in this instance are calculated using the comparison cloud feature in the wordcloud package in R (Fellows, 2018).
